# Different Effects of Alcohol Exposure on Action and Outcome-Related Orbitofrontal Cortex Activity

**DOI:** 10.1523/ENEURO.0052-21.2021

**Published:** 2021-04-15

**Authors:** Christian Cazares, Drew C. Schreiner, Christina M. Gremel

**Affiliations:** 1 The Neurosciences Graduate Program, University of California San Diego, La Jolla, CA 92093; 2 Department of Psychology, University of California San Diego, La Jolla, CA 92093

**Keywords:** action, alcohol, compulsive, dependence, devaluation, orbitofrontal cortex

## Abstract

Alcohol dependence can result in long-lasting deficits to decision-making and action control. Neurobiological investigations have identified orbitofrontal cortex (OFC) as important for outcome-related contributions to goal-directed actions during decision-making. Prior work has shown that alcohol dependence induces long-lasting changes to OFC function that persist into protracted withdrawal and disrupts goal-directed control over actions. However, it is unclear whether these changes in function alter representation of action and outcome-related neural activity in OFC. Here, we used the well-validated chronic intermittent ethanol (CIE) exposure and withdrawal procedure to model alcohol dependence in mice and performed *in vivo* extracellular recordings during an instrumental task in which lever-press actions made for a food outcome. We found alcohol dependence disrupted goal-directed action control and increased OFC activity associated with lever-pressing but decreased OFC activity during outcome-related epochs. The ability to decode outcome-related information, but not action information, from OFC activity following CIE exposure was reduced. Hence, chronic alcohol exposure induced a long-lasting disruption to OFC function such that activity associated with actions was enhanced, but OFC activity contributions to outcome-related information was diminished. This has important implications for hypotheses regarding compulsive and habitual phenotypes observed in addiction.

## Significance Statement

Alcohol dependence produces long-lasting deficits in decision-making, including to actions made with regard to their outcome or consequence. This type of decision-making has been shown to involve orbitofrontal cortex (OFC). Here, using a mouse model of chronic alcohol exposure, we show that chronic alcohol exposure leads to bidirectional changes in activity of OFC neurons depending on the behavioral computation being performed. Mice that underwent previous chronic alcohol exposure showed increased OFC activity during actions for a food reward, but reduced OFC activity during reward-associated periods, and reduced OFC contribution to reward prediction.

## Introduction

Alcohol use disorder (AUD) can result in decision-making deficits that can persist into protracted abstinence. In those suffering from AUDs, these deficits are thought to contribute to habitual and compulsive alcohol-seeking, a persistent vulnerability to relapse, and decrements in daily cognitive function (Stephens and Duka, 2008; Berre et al., 2012; Reich and Goldman, 2015; Le Berre et al., 2017; Sebold et al., 2017; Bickel et al., 2018). With reports of alcohol dependence-induced functional and structural alterations across the cortex (Volkow et al., 1994, 1997; Laakso et al., 2002; Cardenas et al., 2011; Durazzo et al., 2011; Beck et al., 2012; Sjoerds et al., 2013; Thayer et al., 2016), it is highly likely that a broad array of computations normally contributing to efficacious decision-making are also altered. Identifying which computations are disrupted along with any corresponding aberrant activity patterns would offer a starting point for mechanistic investigations into cortical circuit alterations that produce these long-lasting impairments in decision-making.

One such cortical circuit that often shows long-lasting dependence-induced disruptions is the orbitofrontal cortex (OFC). Abstinent AUD patient studies generally report a hypoactive OFC both at baseline and during adaptive decision-making (Volkow et al., 1994, 1997; Boettiger et al., 2007; Sjoerds et al., 2013; Reiter et al., 2016) but also report OFC hyperactivity to stimuli and related approach behaviors (Wrase et al., 2002; Hermann et al., 2006; Reinhard et al., 2015), reminiscent of OFC hyperactivity reported in patients with other psychiatric conditions, including obsessive compulsive disorder (Milad and Rauch, 2012; Pauls et al., 2014; Robbins et al., 2019; Lüscher et al., 2020). This dichotomy of effects suggests that long-lasting perturbations to OFC circuitry induced by alcohol dependence may differentially alter the computations performed by OFC neurons in response to information coming into OFC. Hence, initial investigations into computations performed by OFC during decision-making and their long-lasting disruption in alcohol dependence would provide a framework with which to investigate broader circuit mechanisms contributing to observed OFC dysfunction.

Several lines of evidence implicate the OFC as a key contributor to computations that can contribute to value-based decision-making processes (Fellows, 2007; Wallis, 2007; Gremel and Costa, 2013; Stalnaker et al., 2015; Padoa-Schioppa and Conen, 2017) as well as to compulsive control (Milad and Rauch, 2012; Pauls et al., 2014; Robbins et al., 2019; Lüscher et al., 2020). OFC neurons will modulate firing rate when subjects make a lever press (Gremel and Costa, 2013) and when presented with an appetitive outcome (Rolls et al., 1996; Wallis, 2011). Functional manipulations to OFC activity have generally supported a role for OFC in using outcome-related information to control decision-making (Gremel and Costa, 2013; Rhodes and Murray, 2013; Baltz et al., 2018; Malvaez et al., 2019). Interestingly, increased OFC activity has also been functionally implicated in contributing to compulsive control over behavior (Ahmari et al., 2013; Burguière et al., 2013; Pascoli et al., 2015, 2018), with previous work showing that increased OFC activity and downstream output supports compulsive lever pressing for dopamine neuron stimulation (Pascoli et al., 2018).

Likewise, animal models of alcohol dependence have revealed long-lasting dependence-induced disruptions to OFC-dependent processes, including behavioral flexibility and outcome devaluation (Badanich et al., 2011; Kroener et al., 2012; Lopez et al., 2014; Fernandez et al., 2017; Renteria et al., 2018, 2020). Chronic heavy alcohol consumption in non-human primates, as well as prior chronic alcohol exposure in mice, results in long-lasting changes to OFC intrinsic excitability, synaptic transmission, and increases in dendritic spine density of OFC neurons (Badanich et al., 2013; McGuier et al., 2015; Nimitvilai et al., 2016, 2017; Renteria et al., 2018). Similar to findings in chronically drinking non-human primates (Nimitvilai et al., 2017), previous work has reported that chronic intermittent ethanol (CIE) vapor exposure and withdrawal procedures in mice led to a reduction in OFC excitability (Renteria et al., 2018). CIE-exposed mice also showed an insensitivity to outcome devaluation in protracted withdrawal, characterized as a reduction in goal-directed control and an increased reliance on habitual control over lever pressing for food. Notably, artificially increasing the activity of OFC projection neurons was sufficient to restore sensitivity to outcome devaluation in CIE-exposed mice (Renteria et al., 2018). To this end, the observed dependence-induced deficits in decision-making are hypothesized to include alterations to OFC activity critical for decision-making processes. However, whether this includes alterations to OFC activity during actions and outcome-related epochs is unknown.

Here, we examined CIE exposure-induced disruptions to OFC activity during protracted withdrawal in an instrumental task where actions are made for a food outcome. We used an adapted action contingency task, historically termed action differentiation (Platt et al., 1973; Kuch, 1974; Yin, 2009; Fan et al., 2012). In this task, mice must learn to press and hold a lever down beyond a fixed minimum duration to earn a food reward. The structure of the task allows us to look at OFC activity at the onset, during, and offset of lever presses, as well as during outcome-related epochs. Prior works have found that alcohol-exposed rats and mice show similar acquisition of lever-press performance compared with naive controls, but outcome devaluation and contingency degradation procedures (Corbit et al., 2012; Lopez et al., 2014; Morisot et al., 2019; Renteria et al., 2018; Barker et al., 2020) have shown that such lever pressing is controlled by habitual, instead of goal-directed, processes. Thus, in a subset of mice, we performed outcome devaluation testing procedures after acquisition. Replicating previous findings, we show that air and CIE mice acquire similar lever pressing performance. However, subsequent outcome devaluation testing showed that such lever pressing was under habitual control in CIE mice and goal-directed control in air mice. When we examined OFC activity during behavioral acquisition, we found that prior induction of alcohol dependence led to higher OFC firing rates related to lever-pressing, but reduced firing rates during periods associated with outcome delivery. Decoder analyses on OFC activity showed reduced accuracy to classify outcome related information in CIE-exposed mice compared with controls. Thus, while CIE led to increased activity related to actions, it reduced OFCs normal representation of outcome-related information that may be important for goal-directed decision-making.

## Materials and Methods

### Animals

Male and female C57BL/6J mice (*n* = 15, 9 males, 6 females or non-recording experiments; *n* = 18, 17 males, 1 female for recording experiments) were housed two to five per cage under a 14/10 h light/dark cycle with access to food (Labdiet 5015) and water *ad libitum* unless stated otherwise. C57BL/6J (The Jackson Laboratory) mice were at least six weeks of age before intracranial micro-array implant and at least 52 d of age before vapor procedures or behavioral training. Investigators were not blind to the experimental groups. The Animal Care and Use Committee of the University of California, San Diego approved all experiments and experiments were conducted according to the NIH guidelines.

### Surgical procedures

Animals under isoflurane anesthesia were implanted with a stereotaxically guided fixed micro-array consisting of four-rows of four platinum-plated tungsten electrodes (35-μm tip, Innovative Neurophysiology), with electrodes spaced 150 μm apart, and rows 150 μm apart. The dearth of female mice in the recording study was because of problems with female mice not being able to maintain and carry the electrode implant through CIE procedures and behavioral testing. To maximize targeting of the OFC, arrays were centered at the following coordinates from bregma: A, 2.5 mm; M/L, 1.3 mm; V, 2.0 mm. An additional bilateral craniotomy was made over the posterior cerebellum for placement of screws wrapped with the electrical reference wire attached to the micro-array. After testing, mice were euthanized, and brains extracted and fixed in 4% paraformaldehyde. Micro-array placement was qualified by examining tracts in 50- to 100-μm-thick brain slices under a macro fluorescence microscope (Olympus MVX10). A subset of micro-arrays was dyed with a 25 mg/ml 1,1′-dioctadecyl-3,3,3′,3′-tetramethylindocarbocyanine perchlorate (Dil) solution in 200 proof ethanol (Sigma) for placement verification. All surgical and behavioral experiments were performed during the light portion of the cycle.

### CIE exposure and repeated withdrawal

One to two weeks after micro-array implant surgeries for recording mice, all mice were exposed to four rounds of ethanol vapor or air (Becker and Hale, 1993; Becker and Lopez, 2004; Lopez and Becker, 2005; Griffin et al., 2009; Renteria et al., 2018). Each round consisted of 16 h of vapor exposure followed by an 8-h withdrawal period, repeated for four consecutive days. The CIE procedure is designed to repeatedly induce alcohol withdrawal syndrome after long periods of alcohol exposure, a key criterion in the diagnosis of alcohol dependence (Lopez and Becker, 2005). Ethanol was volatilized by bubbling air through a flask containing 95% ethanol at a rate of 2–3 l/min. The resulting ethanol vapor was combined with a separate air stream to give a total flow rate of ∼10 l/min, which was delivered to the mice housed in Plexiglas chambers (Plas Labs Inc). Mice were not pretreated with a loading dose of ethanol or pyrazole to avoid confounding effects of stress that can bias reliance on habitual control, as well as to avoid the effects of pyrazole on neural activity, including actions at the NMDA receptor (Pereira et al., 1992; Becker and Lopez, 2004; Dias-Ferreira et al., 2009). Blood ethanol concentrations (BECs) were collected at the end of each round from separate, non-experimental mice to avoid previously reported stress effects on decision-making from blood extraction on air mice (mean ± SEM BEC = 29.53 ± 2.36 mm). BEC assays that experienced technical errors were excluded from this measurement.

### Behavioral task

We adapted a lever press hold down task previously used to assay the timing of decision-making actions in mice (Yin, 2009; Fan et al., 2012). Mice were trained in standard operant chambers with one lever extended to the left (or right) of a food magazine and a house light on the opposite wall within sound-attenuating boxes (Med-Associates). Two days before training, mice were food restricted and maintained at 85−90% of their baseline body weight throughout training and testing.

#### Magazine training

On the first day, mice were trained to retrieve pellets from the food magazine (no levers present) on a random time (RT) schedule, with a pellet outcome delivered on average every 120 s for 60 min.

#### Continuous reinforcement

The next 3 d the left (or right) lever was present the entire duration of the session. Lever presses were rewarded on a continuous reinforcement (CRF) schedule for up to 15 (CRF day 1), 30 (CRF day 2), or 60 (CRF day 3) pellet deliveries or until 60–90 min had passed. For electrode-implanted animals, an additional CRF training day (4 d total) was administered with the implant connected to the amplifier board to habituate the animal to the tethered connection.

#### Lever press hold down training

The action differentiation task required lever press durations to exceed a duration criterion assigned before the start of the daily session. This criterion was the minimum duration of time the animal was required to hold the lever in a depressed position to receive a reward. Each session began with the house light turning on and the left (or right) lever being extended for the duration of the session. Lever pressing was self-initiated and self-paced without an imposed trial structure (i.e., the lever was never retracted until the session was complete). Reward delivery occurred at the offset of the lever press only if the hold down timer exceeded the session’s assigned duration criterion. Sessions were completed when 30 outcomes (non-recording animals) or 60 outcomes (recording animals) were earned or after 90 min, whichever came first. The lever press duration criterion for the first 5 d was 800 ms, followed by 5 d (4 d in three animals because of loss of head-cap implant) of a lever press duration requirement of 1600 ms.

#### Devaluation testing

Following the last day of hold down testing in the behavioral cohort, mice were habituated to a novel cage and 20% sucrose solution for 1 h each. Devaluation testing through sensory-specific satiation was conducted across 2 d and consisted of a valued day and a devalued day. For the valued day, the mice were allowed to prefeed for 1 h on 20% sucrose solution. For the devalued day, mice could prefeed for 1 h on the pellet outcome previously earned in the lever press hold down task. Mice that did not consume enough pellets (<0.1 g) or sucrose (<0.1 ml) during prefeeding were excluded from subsequent analysis (CIE cohort, *n* = 1). Each day immediately following prefeeding, mice were placed into their respective operant chamber for 10 min, where the number and duration of lever presses made were recorded, but no outcome was delivered. Investigators were not blind to the experimental groups. Valued and devalued days were counterbalanced and run across consecutive days. Response rate comparisons between valued and devaluated days were made by normalizing each mouse’s test day response rate to the average response rate of their corresponding last 2 d of 1600-ms duration criterion sessions using the following formula:

$$RR_{Test\;\;Day÷}\frac{\;}{RR_{1600-4}+RR_{1600-5}}$$

### Electrophysiological recordings and spike sorting

Spike activity and local field potentials were recorded using an RHD2000 USB interface board system connected to an amplifier board via a serial peripheral interface (SPI) cable (Intan Technologies). Electrode signals were amplified, digitized at 30 kHz and filtered between 0.1 Hz and 6 kHz for spikes and 0.1 and 600 Hz for local field potentials. Initial sorting occurred before each testing session using an online-sorting algorithm (OpenEphys; Siegle et al., 2017). Behavior events that occurred inside the operant boxes were timestamped in synchronization in OpenEphys with neural activity using transistor-transistor logic (TTL) pulses collected at a 10-ms resolution from Med Associates SuperPort Output cards. Spike data were re-sorted offline (Offline Sorter, Plexon) using a T-Distribution Expectation-Maximization Scan algorithm in 3D feature space (Shoham et al., 2003). This allowed for the identification of neuronal activity units based on waveform, amplitude, and inter spike interval histogram (no spikes during a refractory period of 1.4 ms). After sorting, each isolated cluster of waveforms was then manually inspected, and biologically implausible waveform clusters were removed from further analysis. To ensure high signal-to-noise quality of each waveform cluster, waveforms 2 standard deviations (SDs) greater than the clustered population mean were excluded from the analyses. Units with <1000 spike waveforms captured within an entire recording session or that did not show consistent activity across a recording session were not included in our analyses. Before each recording session, mice were exposed to a brief (10–20 s) bout of low-dose isoflurane anesthesia to connect the implant with the recording cable. To avoid confounding effects of anesthesia on brain activity, mice were then moved into the procedure room and monitored for a minimum of 30 min before placing them in the operant chamber and initiating the session.

### Identification of significantly modulated units

To initially examine task-related neural activity, for each previously isolated recorded unit we constructed a peri-event histogram (PETH) around time-stamped lever-press and reward delivery events, such that neural activity was binned into 20-ms bins and averaged across events to analyze amplitude and latency during the recorded behaviors. Per-unit PETHs were then smoothed using a Gaussian-weighted moving average over three bins (60 ms). Using the distribution of the PETH from 10,000 to 2000 ms before lever press onset as baseline activity, we focused our analysis on a period 2000 ms before to 10,000 ms after task-related events. A task-related neuron was up-modulated if it had a significant increase in firing rate defined as at least four bins (80 ms) with a firing rate larger than a threshold of 95% confidence interval above baseline activity during the period from 2000 before to 3000 ms after each task event. A task-related neuron was down-modulated if it had a significant decrease in firing rate if at least four consecutive bins (80 ms) had a firing rate smaller than a threshold of 95% confidence interval below baseline activity during the period from 2000 before to 3000 ms after each task event (Jin and Costa, 2010). The onset of significantly modulated task-related activity was defined as the first of these four-consecutive significant PETH bins. To examine the net effect of CIE on OFC activity as animals performed the task, we combined these up-modulated and down-modulated unit populations for subsequent population analyses.

### Population analyses

#### Performance-related spike activity

To investigate differences in peri-event spike activity between lever presses that were rewarded or not, spike timestamps occurring 10,000 ms before to 10,000 ms after individual lever press events were split into successes (lever press duration exceeded session’s criterion duration) and failures (lever press duration did not exceed session’s criterion duration). Performance segmented neural activity was then binned into 20-ms bins, averaged across events, and then smoothed using a Gaussian-weighted moving average over three bins (60 ms), resulting in two PETHs per unit (successes or failures). Individual PETHs were then converted to z-scores using the mean and SD of the firing rate during a baseline period occurring 10,000–2000 before lever press onset. Per-unit z-scored PETHs were then averaged by treatment group to construct population response profiles for each group. Population spike activity from the last the two sessions of 1600-ms duration criteria was grouped such that a minimum of one session per animal was included. Population spike activity traces were then smoothed with MATLAB’s Savitzky–Golay *smoothdata* method using a 400-sample sliding window for visual display purposes only.

#### Ongoing lever press-related spike activity

To investigate differences in spike activity during ongoing lever-presses, each lever press duration was first calculated by subtracting the lever press onset timestamp from lever press offset timestamp. Each lever press duration was then segmented into four equivalent segment bins (i.e., 0–25%, 25–50%, 50–75%, 75–100% of lever press duration), and all spikes occurring within each of these duration bins were counted and calculated as a proportion of all spikes that occurred during that entire lever press.

To investigate differences in firing rate changes between different lever press durations, lever presses were first grouped into four quartiles determined by the distribution of lever press durations within each individual recording session. Quartile-grouped spike activity occurring 10,000 ms before to 10,000 ms after lever press onset was then binned into 20-ms bins, averaged across lever presses, and then smoothed using a Gaussian-weighted moving average over three bins (60 ms), resulting in four PETHs per unit, one for each duration quartile. To account for variable lever press durations, PETHs were converted to z-scores using the mean and SD of activity occurring before the onset of the lever press proportionate in duration to the average lever press duration within each quartile. Individual lever press activity from these PETHs were then segmented into four equivalent segment bins (i.e., 0–25%, 25–50%, 50–75%, 75–100% of lever press duration). Per-unit, baseline z-scored traces were then averaged across the four duration segments within quartile and treatment groups to construct population response profiles.

#### Neural decoding of task performance from spike activity

For all units from recording sessions in which a minimum of 10 lever presses exceeded the session’s lever press duration criterion, spike timestamps occurring 2000 ms before to 10,000 ms after individual lever press events were binned into 1-ms bins and labeled by lever press outcome (success or failure to exceed the session’s lever press duration criterion). These peri-event rasters were then segmented by treatment groups and task event (lever press onset or offset) and used to train a model to classify successful lever presses. The classifier, a support vector machine model implemented in MATLAB with the NDT toolbox, was trained and tested at 100-ms steps with a bin width of 200 ms (Meyers, 2013). For each of these time points, the classifier used 10 cross-validation splits to segment per-unit firing rates from randomly selected lever press events into training (90%) and testing (10%) sets for 500 resampling runs. Significance at each of these timepoints was tested by first creating 5 null distributions of decoding accuracy with 500 resampling runs each in which the performance labels were shuffled. The accuracy of our decoder was then compared with these null distributions across all time points.

### Statistical procedures

Statistical significance was defined as an α of *p *<* *0.05. Statistical analysis was performed using GraphPad Prism 8.3.0 (GraphPad Software) and custom MATLAB R2019a (MathWorks) scripts using a PC desktop with Windows 10. Acquisition data, including lever presses, response rate, and proportion of lever presses that were rewarded were analyzed using two-way repeated measures ANOVA (session × treatment) unless otherwise noted. For outcome devaluation testing, two-way repeated measure ANOVAs (value × treatment) with preplanned *post hoc* Sidak’s multiple comparison testing were performed to examine whether outcome devaluation reduced lever pressing on the devalued compared with valued day within each group. For peri-event spike activity comparisons, per-unit average z-scored firing rates were binned into four 250-ms bins before the lever press onset, or after lever press offset and after reward delivery, respectively. Within treatment groups, we performed two-way repeated measure ANOVAs (bin × outcome) to examine differences in spike activity between lever presses that failed or succeeded to exceed the session’s lever press duration criteria, with *post hoc* Sidak’s multiple comparison testing to determine bins in which differences were pronounced. Two-way repeated measure ANOVAs (bin x treatment) were performed to examine differences in spike activity between treatment groups, with *post hoc* Sidak’s multiple comparison testing to determine bins in which differences were pronounced. Two-way repeated measure ANOVAs (segment × treatment) were performed to examine group differences in the proportions of spikes occurring between lever press duration segments. Between group comparisons of decoder accuracy were made with two-way repeated measure ANOVAs (bin × treatment), with *post hoc* Benjamini and Hochberg multiple comparison testing to examine in which of the 100-ms bins were differences pronounced. When appropriate, mixed-effect analyses were conducted in lieu of repeated measures ANOVAs (e.g., when data points were missing because of loss of implant). Data are presented as mean ± SEM.

### Code accessibility

The code/software described in the paper is freely available online at https://github.com/gremellab/CIEOFCHOLD. The code is available as Extended Data 1(.m files). All data generated or analyzed during this study are available at https://doi.org/10.6084/m9.figshare.12605225.v1.

10.1523/ENEURO.0052-21.2021.ed1-1 Extended Data 1 Behavioral timestamp extraction, spike activity analysis, decoder classification, and plot creation code (.m files). Download Extended Data 1, ZIP file.

## Results

### Induction of ethanol dependence disrupts goal-directed control over lever pressing

**Figure 1.**
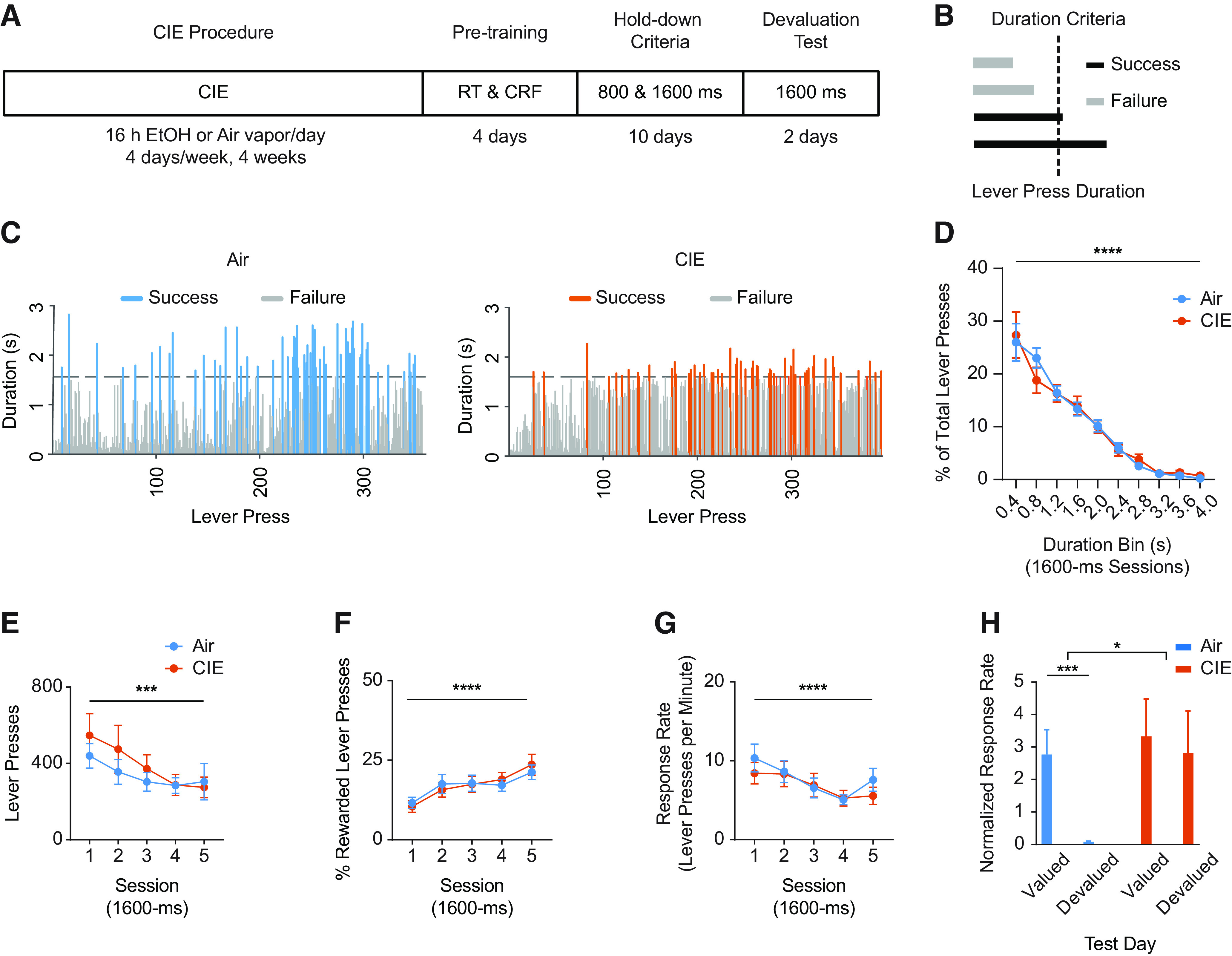
Effects of alcohol dependence on lever press acquisition (***A–G***) and outcome devaluation (***H***). ***A***, Experimental timeline starting with the CIE procedure, subsequent RT delivery of outcome, fixed-ratio CRF on lever press, five daily sessions of 800-ms, and then five daily sessions of 1600-ms, lever press duration criterion sessions and, lastly, two subsequent days of outcome devaluation (DV) testing. ***B***, Schematic of lever press duration performance. Lever presses exceeding the session’s minimum hold-down duration criterion were rewarded only at the offset of the lever press. ***C***, Example lever press performance from individual air (left) and CIE (right) mice during a 1600-ms hold-down duration criterion session late in training. Distribution of lever press durations (***D***), average total lever presses (***E***), average percentage of rewarded lever presses (***F***), and average response rate through 1600-ms lever press duration criterion sessions (***G***). ***H***, Average normalized response rate in valued and devalued states throughout devaluation testing. Lever press duration distributions throughout acquisition and predevaluation test food consumption are shown in Extended Data Figure 1-1. Data points represent mean ± SEM; *p < 0.05, ***p < 0.001, ****p < 0.0001.

We employed a well-validated model of CIE vapor exposure and repeated withdrawal (Becker and Hale, 1993; Becker and Lopez, 2004; Lopez and Becker, 2005; Griffin et al., 2009). Mice were exposed to periods of ethanol (CIE) or air (air) vapor and subsequent withdrawal over a period of four weeks (six vapor cohorts; air: *n* = 17, CIE: *n* = 16; Fig. 1*A*). CIE procedures produced mean BECs in ethanol-exposed mice in line with previous reports (29.53 ± 2.36 mm; Lopez and Becker, 2005; Renteria et al., 2018). Alcohol withdrawal has been delineated into two phases; an immediate acute withdrawal period (2–3 d), followed by a protracted period extending at least three months (Heilig et al., 2010). To examine OFC activity and related behavior during this protracted withdrawal period, food-restricted mice began instrumental training and testing procedures 5 d after their last vapor exposure.

10.1523/ENEURO.0052-21.2021.f1-1 Extended Data Figure 1-1 ***A–C***, Lever press performance throughout acquisition and predevaluation test food consumption. ***A***, Distribution of the percentage of total lever presses binned by duration for 800- and 1600-ms criteria days for air and (***B***) CIE groups. In the air group, a two-way repeated measures ANOVA (bin × criteria) on the lever press duration distribution revealed an interaction: *F*_(9,270)_ = 6.21, *p* < 0.0001, and a main effect of bin: *F*_(9,270)_ = 87.73, *p* < 0.0001 (*post hoc* comparisons, *p* < 0.05). In the CIE group, a two-way repeated measures ANOVA (bin × criteria) on the lever press duration distribution revealed an interaction: *F*_(9,261)_ = 4.37, *p* < 0.0001, and a main effect of bin only: *F*_(9,261)_ = 57.03, *p* < 0.0001 (*post hoc* comparisons *p* < 0.05). ***C***, Total grams of food pellet and 20% sucrose solution consumed during each 1-h *ad libitum* access feeding period before each 10-min outcome devaluation test session. A two-way repeated measures ANOVA (food type × treatment) on grams consumed during this period found no interaction, but a main effect of food type: *F*_(1,20)_ = 25.2, *p* > 0.0001. Data points represent mean ± SEM; **p < 0.01, ***p < 0.001, ****p < 0.0001. Download Figure 1-1, EPS file.

To examine the effects of prior CIE procedures on OFC activity during action and outcome-related epochs in decision-making, we adapted an instrumental task examining action differentiation (Fig. 1*A*), where a mouse must learn to press and hold a lever down beyond a fixed minimum duration to earn a reward (Yin, 2009; Fan et al., 2012). Throughout training, mice learned to press and hold down a lever beyond a predetermined minimum duration to earn a food pellet on release of the lever. Mice self-initiated and self-terminated every lever press in the absence of any extrinsic cues signaling lever press duration. Importantly, reward delivery occurred only at the offset of a lever press that exceeded the duration criteria (Fig. 1*B*,*C*), preventing the use of reward presence to signal lever press termination. Thus, this task produces discretized behavior epochs conducive to neural activity analysis (e.g., lever-press onset, during the lever-press, lever-press offset, and outcome delivery).

Following vapor procedures, air and CIE mice underwent action differentiation training, with the initial criterion for lever press duration set at 800-ms for five daily sessions, followed by five daily sessions of a 1600-ms criterion (see Materials and Methods). Representative sessions from one air and one CIE mouse on a 1600-ms criteria day are shown in Figure 1*C*, suggesting that mice show a distribution of lever press durations that approximate the duration criterion. This distribution of lever presses was similar between air and CIE mice (1600-ms criteria training sessions, two-way repeated measures ANOVA (bin × treatment); no interaction; main effect of bin: *F*_(9,252)_ = 53.56, *p* < 0.0001), and importantly, each group showed evidence of learning with rightward shifts in their corresponding lever press duration distributions following the switch from 800- to 1600-ms training criteria (Extended Data Fig. 1-1*A*,*B*). We focused our examinations on behavior throughout the 1600-ms criterion sessions, after mice had learned the action differentiation rule and had shifted to a longer duration contingency. Air and CIE mice showed similar levels of lever pressing that increased across sessions (mixed-effects repeated measures ANOVA (session × treatment); no interaction; main effect of session only: *F*_(4,121)_ = 5.36, *p* < 0.0001; Fig. 1*E*). This task was similarly challenging for air and CIE mice, and only ∼25% of total lever presses in air and CIE mice exceeded the duration criterion within each session (mixed-effects repeated measures ANOVA (session × treatment); no interaction; main effect of session: *F*_(4,121)_ = 12.28, *p* < 0.0001; Fig. 1*F*). Further, CIE treatment did not alter response rates, which increased across sessions for both air and CIE mice (mixed-effects repeated measures ANOVA (session × treatment); no interaction; main effect of session: *F*_(4,121)_ = 12.28, *p* < 0.0001). Thus, as expected, CIE and air mice show similar lever press acquisition for food reward.

Examination of lever press performance as discussed above, however, cannot distinguish whether lever press is under goal-directed of habitual control. To examine whether the observed lever pressing was under different action controllers in CIE compared with air mice, we performed outcome devaluation procedures and testing following training on the 1600-ms duration criterion. In outcome devaluation testing, a reduction in response rates following prefeeding with the outcome normally earned through lever pressing has been defined as a measure of goal-directed control (Dickinson, 1985). Prior work has found that alcohol dependence reduces the contribution of goal-directed control to lever pressing for food (Dickinson, 1985; Lopez et al., 2014; Renteria et al., 2018) within the time frame examined in the present experiment (Renteria et al., 2018). We subjected a subset of air and CIE mice to sensory-specific satiation of food pellets previously earned by lever pressing or to a previously habituated control outcome (20% sucrose solution). In each of the two consecutive test days, mice had *ad libitum* access to either pellets (devalued state) or sucrose solution (valued state) for 1 h before measuring non-reinforced lever press responses in the operant chamber throughout each 10-min 1600-ms duration criterion session. While air mice clearly reduced response rates (normalized to the response rate during last two days of acquisition) in the devalued state compared with valued state, CIE mice did not. A two-way repeated measures ANOVA (value × treatment) revealed different patterns of responding in treatment groups (interaction: *F*_(1,22)_ = 6.961, *p* = 0.015; main effect of value only: *F*_(1,22)_ = 12.54, *p* = 0.001). *Post hoc* analysis on normalized response rates revealed a difference between valued and devalued test sessions in air mice (*p* = 0.0002), but not in CIE mice (*p* > 0.5). As air and CIE mice consumed similar amounts of both outcomes during the prefeeding periods (Extended Data Fig. 1-1*C*), the data show that lever press performance in CIE mice was not controlled by goal-directed processes.

### OFC populations differentially encode lever-pressing and outcome-related components

**Figure 2.**
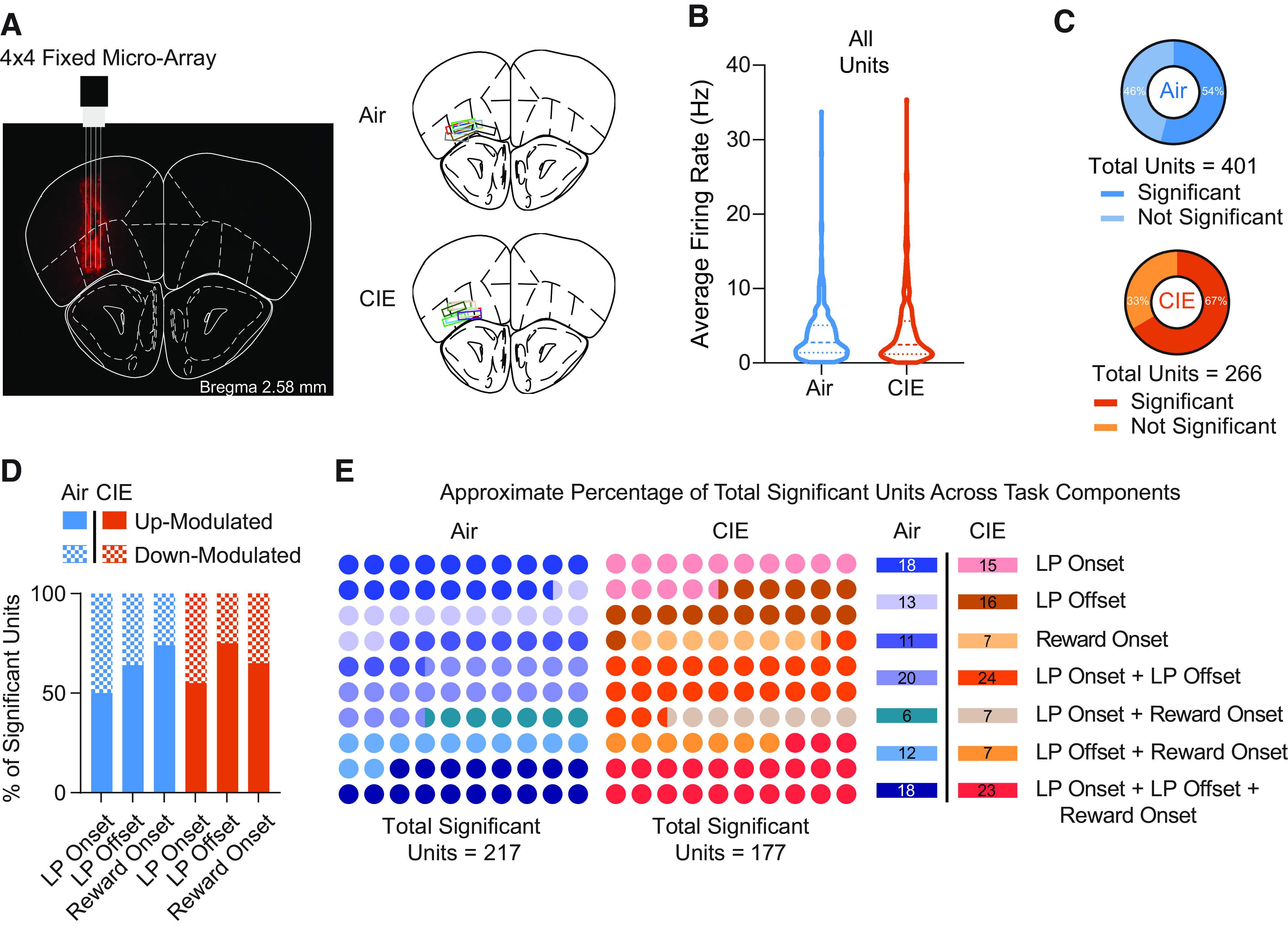
***A–E***, OFC activity correlates of lever pressing and outcome delivery. ***A***, Representative image of fixed micro-array implant over the orbitofrontal cortex. Implant locations segmented by air and CIE groups. A subset of micro-arrays was dyed with a 25 mg/ml Dil solution in 200 proof ethanol for placement verification. ***B***, Average firing rates from all captured units during a baseline period (−5 to −2 s before lever press onset) for air and CIE cohorts. ***C***, Proportion of all captured units in which firing rates significantly deviated from a baseline period (−5 to −2 s before lever press onset) for air (∼54%) and CIE (∼66%) groups. ***D***, Percentage of units that significantly increased or decreased their firing rates from baseline in relation to lever press onset, offset, and food pellet reward delivery. ***E***, Percentage of units that significantly changed their firing rates from baseline in relation to discrete task events (lever press onset: air: ∼18%, CIE: ∼15%; lever press offset: air: ∼13%, CIE: ∼16%; reward delivery: air: ∼11%, CIE: ∼7%) as well as multiple task components (lever press onset and offset: air: ∼20%, CIE: ∼24%; lever press onset and reward delivery: air: ∼6%, CIE: ∼7%; lever press offset and reward delivery: air: ∼12%, CIE: ∼7%; lever press onset, offset, and reward delivery: ∼18%, CIE: ∼23%).

Given that the deficits in outcome devaluation observed in CIE mice have been shown to involve OFC (Gourley et al., 2013; Gremel and Costa, 2013; Rhodes and Murray, 2013; Gremel et al., 2016; Renteria et al., 2018), and OFC modulates activity during outcome-related epochs (Rolls et al., 1996; Wallis, 2011), we hypothesized that alcohol exposure would disrupt OFC neural activity related to lever press and outcome-related epochs in our task. We examined OFC activity in relation to task epochs in a subset of the mice that had been implanted with chronic indwelling micro-electrode arrays into the OFC before the start of CIE procedures (five vapor cohorts; air *n* = 9, CIE *n* = 9; Fig. 2*A*).

We focused on OFC activity data collected during the last two sessions of the 1600-ms duration criterion, a time point during which animals from both groups most proficiently performed the task. Putative single OFC unit spike activity was aligned to timestamps collected each time a lever press onset, offset, or pellet reward delivery occurred (see Materials and Methods). Importantly, there was no effect of CIE exposure on average baseline firing rates (*p* > 0.05; Fig. 2*B*). More OFC units in CIE mice (67%) than air mice (54%) showed significantly altered firing rates during any task-related epochs (Fig. 2*C*; χ^2^_1,667_ = 10.21, *p* < 0.002; see Materials and Methods). However, in both groups, we found similar proportions of OFC units that significantly up-modulated (increased firing rate) or down-modulated (decreased firing rate) across lever press onset, lever press offset, and outcome delivery epochs (χ^2^s < 3.76, *p*s > 0.05; Fig. 2*D*). Furthermore, we found that in both air and CIE mice, individual OFC units usually altered activity across multiple epochs (e.g., both the onset and offset of a lever press; Fig. 2*E*; χ^2^_6,394_ = 8.04, *p* < 0.24), with relative high percentages of OFC units encoding action onset, action offset, and reward (air = 18%; CIE = 23%). Air and CIE mice showed similar numbers of significantly modulated units/mouse across events (lever press onset (air = 7.83 ± 1.8, CIE = 7.3 ± 1.8); lever press offset (air = 8.06 ± 1.6, CIE = 7.11 ± 1.7), reinforcement delivery (air = 6.11 ± 2.3, CIE = 4.61 ± 1.33). Altogether, this suggests that the OFC populations normally recruited during this instrumental task were largely not altered following the induction of alcohol dependence.

### Prior CIE procedures enhances OFC lever press-related activity

**Figure 3.**
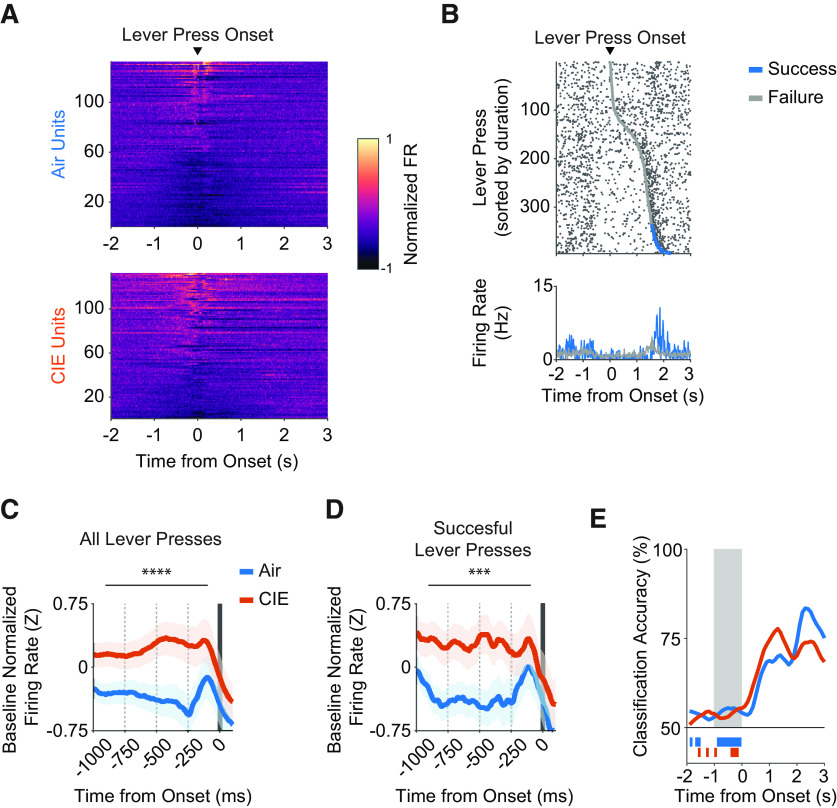
***A–E***, Ethanol dependence alters OFC activity correlates of lever pressing initiation. ***A***, Heat map of normalized firing rates for units that significantly increased or decreased from baseline, displayed relative to lever press onset. Units are sorted by activity from a 1-s window around lever press onset. ***B***, Raster plot of a representative unit’s firing rate relative to lever press onset, sorted from shortest to longest durations within a 1600-ms lever press criterion session that occurred late in training. Gray and blue markers indicate the end of lever presses that failed or succeeded in exceeding the 1600-ms lever press criterion, respectively. ***C***, Average z-scored firing rate changes from baseline for all lever presses and (***D***) successful lever presses only. Firing rate changes were compared across four 250-ms bins relative to lever press onset. ***E***, SVM classification accuracy of task performance outcomes (i.e., did lever press exceed 1600-ms hold-down criterion?) from all captured air and CIE units, displayed relative to lever press onset. Bars underneath traces indicate time points before the onset of the lever press in which classification accuracy was significantly different compared with the null distribution. Shaded region indicates time points in which classification accuracy comparisons were made between air and CIE groups. OFC activity correlates of lever pressing initiation are shown by significantly up-modulated and down-modulated unit averages in Extended Data Figure 3-1. Data points represent mean ± SEM; ***p < 0.001, ****p < 0.0001.

While we observed similar recruitment of OFC populations during behavior, it may be that the magnitude and patterns of OFC activity during task-related epochs are different between the two groups. We first asked whether CIE would alter OFC activity associated with the initiation of lever pressing. We examined the firing rate activity of all significantly modulated units during the 1000-ms period preceding lever press onset, as shown in the normalized activity peri-event heatmaps in both air and CIE animals (Fig. 3*A*). We found greater increases in baseline normalized OFC firing rates before the onset of lever pressing in CIE mice compared with air mice (Fig. 3*C*). A two-way repeated measures ANOVA (250-ms bin × treatment) showed no interaction, but a main effect of treatment only (*F*_(1,1032)_ = 17.39, *p* < 0.0001). This increase in firing rates was also present when we examined up-modulated and down-modulated CIE populations separately (Extended Data Fig. 3-1*A*,*B*), suggesting an overall increase in action-related OFC activity in CIE mice.

10.1523/ENEURO.0052-21.2021.f3-1 Extended Data Figure 3-1 ***A–D***, OFC activity correlates of lever pressing initiation are altered by ethanol dependence but do not reflect future outcomes. ***A***, Average z-scored firing rate changes from all lever presses for significantly up-modulated and (***B***) down-modulated units from air and CIE groups, displayed relative to lever press onset. In up-modulated units, a two-way repeated measures ANOVA (bin × treatment) on activity prior to lever press onset revealed no interactions, but main effects of bin: *F*_(3,564)_ = 12.34, *p* < 0.0001 and treatment: *F*_(1,564)_ = 5.878, *p* = 0.016. In down-modulated units, a two-way repeated measures ANOVA (bin × treatment) on activity prior to lever press onset revealed no interactions, but main effects of bin: *F*_(3,508)_ = 25.11, *p* < 0.0001 and treatment: *F*_(1,508)_ = 38.84, *p* < 0.0001. *Post hoc* comparisons on down-modulated unit activity found differences between air and CIE groups in the 2nd (*p* = 0.036), 3rd (*p* < 0.001), and 4th (*p* = 0.0045) bins. ***C***, Air (left) and CIE (right) group’s average z-scored firing rate changes of up-modulated and (***D***) down-modulated units, segmented by whether a lever press successfully exceeded the 1600-ms duration criterion or not, and displayed relative to lever press onset. For the air group, individual two-way repeated measures ANOVAs (bin × outcome) on activity prior to lever press onset revealed no interactions, but main effect of bin only for up-modulated (*F*_(3,560)_ = 14.47, *p* < 0.0001) and down-modulated (*F*_(3,552)_ = 37.13, *p* < 0.0001) units. Similarly, for the CIE group, individual two-way repeated measures ANOVAs (bin × outcome) on activity prior to lever press onset revealed no interactions, but main effect of bin only for up-modulated (*F*_(3,568)_ = 4.19, *p* = 0.006) and down-modulated (*F*_(3,464)_ = 9.77, *p* < 0.0001) units. Average z-scored firing rate changes were compared between treatment groups or outcomes across four 250-ms bins relative to lever press initiation. Data points represent mean ± SEM; *p < 0.05, **p < 0.01, ***p < 0.001, ****p < 0.0001. Download Figure 3-1, EPS file.

We next asked whether activity associated with lever press initiation reflected future performance outcomes, i.e., were firing rates different for lever presses that were eventually rewarded? To this end, we grouped lever press durations by whether they successfully exceeded the lever press duration criterion or not (Fig. 3*B*). We did not find evidence of predictive coding of successful performance in either air or CIE mice (Extended Data Fig. 3-1*C*,*D*). In addition, when we examined only lever presses that were successful, the increased firing rate observed in CIE mice was still present (two-way repeated measures ANOVA (bin × treatment): main effect of treatment only (*F*_(1,1032)_ = 14.58, *p* = 0.0001; Fig. 3*D*). We then trained a support vector machine (SVN) model with the peri-event firing rate activity of all captured (including significant and non-significant activity modulation in relation to behavioral epochs) units to directly test whether firing rates could accurately classify whether an individual lever press exceeded the 1600-ms duration criterion. In line with the lack of predictive coding, we found overall low classification performance that did not differ between air and CIE mice. A two-way repeated measures ANOVA (bin × treatment) comparisons of temporally binned classification performance between air and CIE mice revealed no significant differences within the 1000-ms period preceding lever press onset (*p* > 0.29). Thus, prior CIE procedures increased OFC activity associated with action onset; however, this activity, as well as activity in air control mice, was not predictive of impending lever-press success.

### Prior CIE procedures have little effect on OFC activity during lever press execution

We next asked whether the increased OFC activity observed in CIE mice before lever-press onset would persist as mice held down the lever. Our analysis focused solely on units that were significantly modulated before the onset of a lever press. When we examined individual unit activity raster plots (Fig. 3*B*), we often observed broad reductions in OFC activity as mice held down the lever. Indeed, we found on average only ∼38% of total lever presses made had at least one putative action potential occur during lever press execution, and this was not different in CIE mice (air mice = 37.85 ± 2%; CIE mice = 38.25± 2%; unpaired *t* test with Welch’s correction: *t*_(257.4)_ = 0.15, *p* = 0.88).

**Figure 4.**
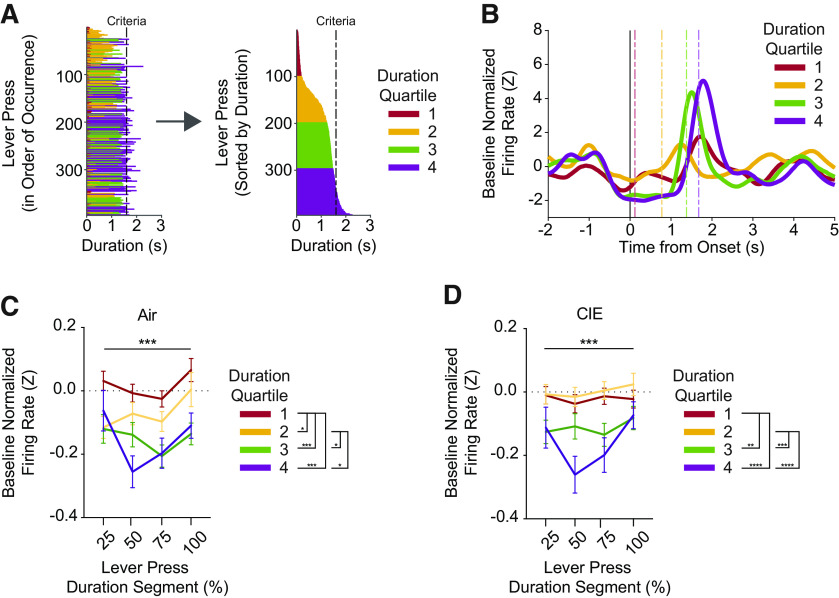
***A–D***, OFC reduces firing while mice hold down the lever. ***A***, Lever presses were segmented into four quartiles determined by the distribution of lever press durations within each individual session. ***B***, Representative unit’s mean baseline normalized z-scored firing rate changes displayed relative to lever press onset. Dashed lines indicate the mean lever press duration for each respective duration quartile within the session. ***C***, Mean baseline normalized z-scored firing rate changes across equidistant duration segments (i.e., 0–25%, 25–50%, 50–75%, 75–100% of lever press duration) of lever presses belonging to each duration quartile for air and (***D***) CIE groups. Quartile boundaries determined by within-session lever press duration distributions are shown in Extended Data Figure 4-1. Data points represent mean ± SEM; *p < 0.05, **p < 0.01, ***p < 0.001, ****p < 0.0001.

As there was a distribution of lever press durations in each session, we next examined whether this relative reduction in OFC activity was different depending on the duration of the lever press being executed. We also examined whether prior CIE procedures would alter any potential change in activity during the lever press itself. To examine this, for a given mouse on a given day, we first divided lever presses within a session into four quartiles (see Materials and Methods; Fig. 4*A*). Mean quartile distribution boundaries were similar between groups (Extended Data Fig. 4-1*A*,*C*). Then, for each of the lever presses made in those quartiles, spike activity that happened while the mouse was holding down the lever (duration of the press) was z-scored normalized to baseline and divided into four equal segments spanning the lever press duration. This segment z-scored activity was then averaged within air and CIE groups. As shown by baseline normalized z-scored activity from a representative unit in Figure 4*B*, this allowed us to examine activity changes across the duration of the lever press based on the relative length of the final lever press duration.

10.1523/ENEURO.0052-21.2021.f4-1 Extended Data Figure 4-1 ***A–C***, Quartile boundaries were determined by within-session lever press duration distributions and do not differ between air and CIE groups. ***A***, Examples session in which lever press durations were binned into four quartiles determined by the distribution of lever press durations. ***B***, ***C***, Duration quartile bin boundaries were similar between groups across the last two 1600-ms hold down criteria sessions that were included in our firing rate analyses. Dashed lines indicate 1600-ms duration criterion. For the starting edges of these bin boundaries, a two-way ANOVA (quartile × treatment) found no interaction, but a main effect of quartile only: *F*_(3,136)_ = 226.5, *p* < 0.0001). For the ending edges of these bin boundaries, a two-way ANOVA (quartile × treatment) found no interaction, but a main effect of quartile only: *F*_(3,136)_ = 141.8, *p* < 0.0001). Data points represent mean ± SEM; ****p < 0.0001. Download Figure 4-1, EPS file.

We found OFC firing rates during the execution of the lever press did differ depending on the duration of the lever press; however, prior CIE exposure had very little effect on these patterns. As exemplified by Figure 4*B*, longer lever presses showed lower firing rates during the lever press, with an increase in firing rate occurring close to the release of the lever press. In air mice, a two-way repeated measures ANOVA (quartile × segment) revealed a main effect of quartile (*F*_(3,2176)_ = 15.87, *p* < 0.0001), a main effect of segment (*F*_(3,2176)_ = 4.18, *p* < 0.005), but no interaction. Follow-up main effect analyses on quartile effects showed that baseline normalized averaged z-scored activity within a quartile largely differed from activity in other quartiles. The exception being activity in the longest two quartiles which did not differ from each other (Fig. 4*C*). The same analyses in CIE mice showed similar differences (main effect of quartile: *F*_(3,1952)_ = 15.19, *p* < 0.0001), but no effect of segment or interaction (*p* > 0.05). In addition, air mice also had similar baseline activity during the first and second (i.e., the shortest lever presses) quartiles (Fig. 4*D*). Follow-up analyses on main effects of segments showed few differences, except in air mice where the last quarter of the lever press differed from the preceding quarter (*p* = 0.01), as represented by the increase in firing rate during the last portion of a lever press in Figure 4*B*. Together, the above data suggest that OFC overall reduces its firing rate activity during the execution of the lever press in a manner that reflects the future duration, with longer lever presses showing greater reductions in firing rate, and that CIE does not drastically alter the ability of OFC to do so.

### Prior CIE procedures decrease OFC activity during outcomes

**Figure 5.**
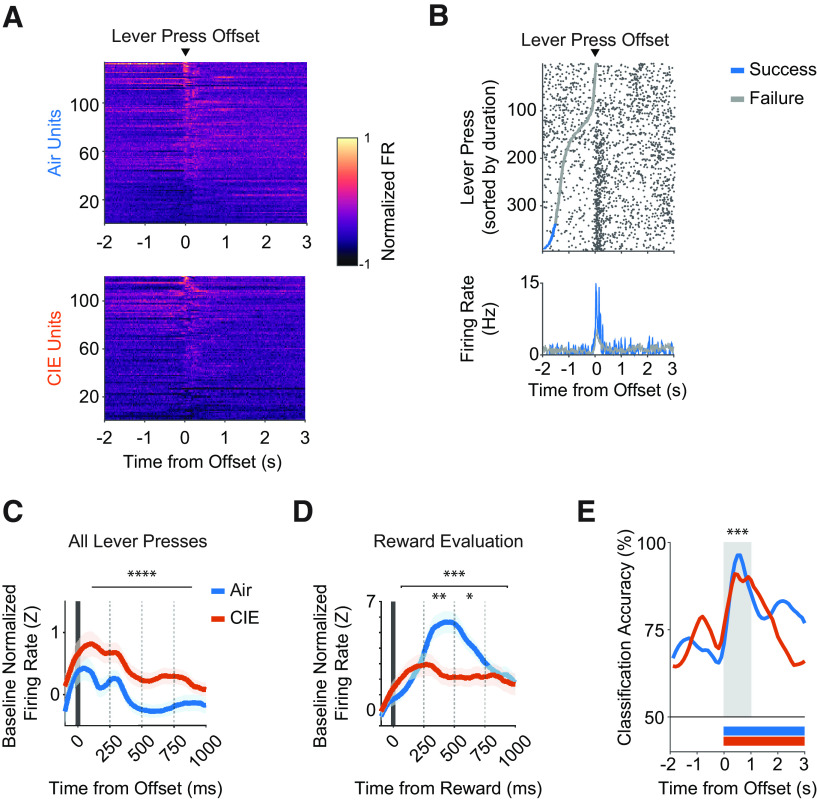
***A–E***, Ethanol dependence alters OFC activity correlates of outcome delivery. ***A***, Heat map of normalized firing rates for units that significantly increased or decreased from baseline, displayed relative to lever press offset. Units are sorted by activity from a 1-s window around lever press offset. ***B***, Raster plot of a representative unit’s firing rate relative to lever press offset, sorted from shortest to longest durations within a 1600-ms lever press criterion session that occurred late in training. Gray and blue markers indicate the start of a lever presses that failed or succeeded in exceeding the 1600-ms lever press criterion, respectively. ***C***, Average z-scored firing rate changes from baseline for all lever presses and (***D***) rewarded lever presses only. Firing rate changes were compared across four 250-ms bins relative to lever press offset. ***E***, SVM classification accuracy of task performance outcomes (i.e., did lever press exceed 1600-ms hold-down criterion?) from all captured air and CIE units, displayed relative to lever press offset. Bars underneath traces indicate time points after the offset of the lever press in which classification accuracy was significantly different compared with the null distribution. Shaded region indicates time points in which classification accuracy comparisons were made between air and CIE groups. OFC activity correlates of lever pressing termination and reward delivery are shown by significantly up-modulated and down-modulated unit averages in Extended Data Figure 5-1. Data points represent mean ± SEM; *p < 0.05, **p < 0.01, ***p < 0.001.

OFC neurons have long been reported to increase their activity in anticipation of and during outcome delivery (Wallis, 2007; Stalnaker et al., 2014, 2018). In the present task, reward delivery cannot occur until the lever is released. Thus, we defined an action offset epoch (1000 ms), and in some cases an outcome-related epoch (3000 ms) following a reward delivery, that encompassed moving to the food receptacle and potentially reward consumption. As the reward is readily visible without mice having to insert their heads into the food receptacle, it is likely that reward perception happens earlier than consumption. As seen in the normalized activity peri-event heatmaps (Fig. 5*A*) and in an example from a representative OFC unit (Fig. 5*B*), OFC firing rates changed significantly during the action offset and outcome-related epochs of the task. We observed similar percentages of OFC units recorded between air and CIE mice where significant activity changes were associated with action offset only (air = 13.5%, CIE = 16.5%) and outcome-related only (air = 11.5%, CIE = 7.5%), as well as OFC units that had activity associated with both action offset and outcome evaluation (air 12%, CIE 7%) or action onset, action offset, and outcome-related (air = 18%; CIE = 23%; Fig. 2*E*).

10.1523/ENEURO.0052-21.2021.f5-1 Extended Data Figure 5-1 ***A–F***, OFC activity correlates of outcome delivery are altered by ethanol dependence and reflect successful performance. ***A***, Average z-scored firing rate changes from all lever presses for significantly up-modulated and (***B***) down-modulated units from air and CIE groups, displayed relative to lever press offset. In up-modulated units, a two-way repeated measures ANOVA (bin × treatment) on activity prior to lever press offset revealed no interactions, but main effects of bin: *F*_(3,748)_ = 21.82, *p* < 0.0001 and treatment: *F*_(1,748)_ = 8.159, *p* = 0.004. In down-modulated units, a two-way repeated measures ANOVA (bin × treatment) on activity prior to lever press offset revealed no differences (*p* > 0.08). ***C***, Average z-scored firing rate changes from rewarded lever presses for significantly up-modulated and (***D***) down-modulated units from air and CIE groups, displayed relative to reward delivery. In up-modulated units, a two-way repeated measures ANOVA (bin × treatment) on activity prior to reward delivery revealed an interaction: *F*_(3,532)_ = 4.508, *p* = 0.004, and a main effect of bin: *F*_(3,532)_ = 7.199, *p* < 0.0001. *Post hoc* comparisons on up-modulated unit activity found differences between air and CIE groups in the 2nd (*p* < 0.03) bin. In down-modulated units, a two-way repeated measures ANOVA (bin × treatment) on activity prior to reward delivery revealed no differences (*p* > 0.2). ***E***, Air (left) and CIE (right) group’s average z-scored firing rate changes of up-modulated and (***F***) down-modulated units, segmented by whether a lever press successfully exceeded the 1600-ms duration criterion or not, and displayed relative to lever press offset. For up-modulated units, individual two-way repeated measures ANOVAs (bin × outcome) on activity after to lever press offset revealed no interactions, but main effects of bin: *F*_(3,736)_ = 5.406, *p* = 0.0011, and outcome: *F*_(1,736)_ = 46.77, *p* < 0.0001 for the air group and no interactions, but main effects of bin: *F*_(3,760)_ = 8.816, *p* < 0.0001, and outcome: *F*_(1,760)_ = 31.08, *p* < 0.0001 for the CIE group. For down-modulated units, individual two-way repeated measures ANOVAs (bin × outcome) on activity after to lever press offset revealed an interaction: *F*_(3,408)_ = 3.916, *p* = 0.009, and main effects of bin: *F*_(3,408)_ = 4.523, *p* = 0.004, and outcome: *F*_(1,408)_ = 81.83, *p* < 0.0001 for the air group, and an interaction: *F*_(3,248)_ = 3.412, *p* = 0.02, and main effects of bin: *F*_(3,248)_ = 4.540, *p* = 0.004, and outcome: *F*_(1,248)_ = 12.79, *p* = 0.0004 for the CIE group. *Post hoc* comparisons on down-modulated unit activity found differences between successful and failed lever presses in the 2nd (*p* < 0.0001), 3rd (*p* < 0.0001), and 4th (*p* = 0.0002) bins for the air group and in the 4th (*p* = 0.0002) bin only for the CIE group. Average z-scored firing rate changes were compared between treatment groups or outcomes across four 250-ms bins relative to the end of the lever press or reward delivery. Data points represent mean ± SEM; *p < 0.05, **p < 0.01, ***p < 0.001, ****p < 0.0001. Download Figure 5-1, EPS file.

When we examined modulation of OFC activity following lever press offset, we found that CIE mice showed greater increases in baseline normalized z-scored OFC firing rates compared with air controls (two-way repeated measures ANOVA (bin × treatment); no interaction; main effect of bin: *F*_(3,1044)_ = 6.612, *p* = 0.0002; main effect of treatment: *F*_(1,1044)_ = 22.63, *p* < 0.0001; Fig. 5*C*; Extended Data Fig. 5-1*A*,*B*). We also found that OFC activity changes at lever press offset reflected performance outcomes in both air and CIE mice (Extended Data Fig. 5-1*E*,*F*); however, it is important to note that this OFC activity was comprised of all lever presses, including those that were rewarded. Thus, we examined OFC firing rate changes aligned to outcome-related epochs following only successful lever presses and asked whether CIE procedures would change the magnitude of these increases. We found large increases in OFC firing rate changes during outcome-related epochs. In contrast to the increase in activity related to lever-pressing in CIE mice, we found air mice had greater increases in OFC firing rates during outcome-related epochs compared with CIE mice (two-way repeated measures ANOVA (bin × treatment); interaction: *F*_(3,724)_ = 4.04, *p* = 0.007; main effect of bin: *F*_(3,724)_ = 4.43, *p* = 0.004; main effect of treatment: *F*_(1,724)_ = 8.66, *p* = 0.003; Fig. 5*D*; Extended Data Fig. 5-1*C*,*D*). Given the overall robust increases in OFC activity during outcome-related epochs, as well as the differences in magnitude induced by CIE procedures, we asked whether a support vector machine (SVM) model trained with the peri-event activity of all captured OFC units could accurately classify whether an individual lever press exceeded the 1600-ms duration criterion during the action offset and outcome-related epochs. We found high classification accuracy during the outcome-related epochs, especially within the first 1000 ms of reward delivery. Furthermore, classification accuracy was lower in OFC units from CIE mice during this period (two-way repeated measures ANOVA (bin × treatment); interaction: (*F*_(9,180)_ = 3.33, *p* = 0.0009; main effect of bin: *F*_(9,180)_ = 5.11, *p* < 0.0001). A *post hoc* Benjamini and Hochberg multiple comparison test revealed that decoder accuracy differences were pronounced in the 1st (*p* = 0.005), 3rd (*p* = 0.006), and 4th (*p* = 0.0086) 100-ms bins of the outcome related epoch. Thus, CIE mice show greater OFC activity associated with lever pressing, but reduced OFC activity during outcome-related epochs, with OFC activity being less predictive of rewarded lever presses.

## Discussion

Alcohol dependence is associated with impairments to OFC function and aberrant decision-making, thereby increasing the vulnerability to relapse and maladaptive alcohol consumption (Zinn et al., 2004; Chanraud et al., 2007; Loeber et al., 2009; Berre et al., 2012; Reiter et al., 2016; Le Berre et al., 2017). Here, we uncovered neural correlates of actions and outcomes and found them perturbed by prior chronic alcohol exposure and withdrawal. Our results suggest alcohol exposure induces long-lasting perturbations to OFC activity in a bidirectional manner, dependent on the computation being performed. CIE mice showed a modulation of OFC activity that suggests overall increases in OFC activity associated with actions (i.e., lever press onset and offset), and a blunting of OFC activity during outcome-related epochs. This raises the hypothesis that alcohol dependence does not result in a loss of OFC recruitment, but rather induces a change in how computations performed by OFC circuits may contribute to decision-making.

In the present data, CIE-exposed animals acquired and performed the lever pressing at similar levels compared with alcohol naive controls (Fig. 1). However, outcome devaluation testing showed that such lever pressing was not under goal-directed control as it was in air mice. Thus, CIE mice were able to acquire lever pressing for food relying on neural mechanisms supporting habit learning. Recent findings corroborate the observed lack of goal-directed control in CIE mice (Lopez et al., 2014; Renteria et al., 2018, 2020; Barker et al., 2020) and with the disruption to decision-making control observed under different instrumental tasks and varied tests of goal-directed control. Our work adds to an ever-growing body of research on such decision-making deficits and highlights the importance of examining alcohol-induced alterations to neural circuits and mechanisms controlling goal-directed processes.

Clinical studies have previously shown that alcohol dependence alters representation of decision-making within OFC circuits, albeit not always in the same manner. The OFC is widely found to be hypoactive in alcohol dependence (Volkow et al., 1994, 1997; Boettiger et al., 2007; Sjoerds et al., 2013; Reiter et al., 2016), but there have also been reports of hyperactivity (Wrase et al., 2002; Tapert et al., 2003; Myrick et al., 2004, 2008; Hermann et al., 2006; Ernst et al., 2014; Reinhard et al., 2015). We find that to be the case in our data as well, such that alcohol exposure altered decision-making representations in the OFC in a variety of ways. Prior CIE exposure and withdrawal changed OFC computations in a way that suggests an overall increase in activity during actions (Fig. 3). This dependence-induced change could suggest an increased contribution of OFC processes to action-related processes. We should emphasize that the action contingency in the present task is the duration of the lever press, and that it is inferred from prior experience. This suggests that alcohol dependence increases OFC activity related to the retrieval and execution of inferred action association. In this context, it is important to note that a hyperactive OFC has also been observed in those with obsessive compulsive disorder and increased activity of OFC neurons during actions has been linked to compulsive action phenotypes (Milad and Rauch, 2012; Pauls et al., 2014; Robbins et al., 2019; Lüscher et al., 2020). Whether this increased OFC activity related to actions that we find in CIE mice plays a role in compulsive phenotypes in alcohol dependence is not currently known.

A hallmark of OFC function is its contribution to reward evaluation and updating, with increases in OFC activity observed during outcome anticipation and presentation (Rolls et al., 1996; Wallis, 2011; Jones et al., 2012; Stalnaker et al., 2014, 2015, 2018). Recent works have shown that inhibition of OFC activity during periods of outcome presentation prevent outcome evaluation and updating (Baltz et al., 2018; Malvaez et al., 2019). Further, recent work in humans has suggested that OFC encodes reward identity expectations (Howard and Kahnt, 2018), which contribute to the generation of prediction errors even when there is no change in value (Stalnaker et al., 2018). Here, we observed large increases in OFC activity during outcome-evaluation periods and this increase was reduced following alcohol exposure (Fig. 5). SVM modeling showed reduced accuracy in classifying rewarded lever presses following alcohol exposure. Furthermore, CIE affected OFC computations made only after successful lever presses. Thus, in addition to action-associated OFC activity, the data above strongly suggests that OFC’s contribution to outcome retrieval, evaluation, or identification is altered following alcohol exposure. Further, results of from decoder analysis showed that OFC activity during outcome-related epochs normally carries information about whether the lever press was successful or not. CIE reduced this OFC representation. Combined with the insensitivity to outcome devaluation, our data support the hypothesis that alcohol dependence leads to reduced contribution of OFC to outcome-related decision-making. Further, the observed bidirectional alcohol exposure effects on OFC computations support the hypothesized complexity of alcohol dependence effects on OFC decision-making circuitry. For instance, previous accounts on the modulatory influence of alcohol dependence on OFC activity have differed (Volkow et al., 1994, 1997; Boettiger et al., 2007; Sjoerds et al., 2013; Reiter et al., 2016), which in conjunction with our findings suggests a divergent effect of alcohol dependence that may be dependent on decision-making demands and information in OFC. While the critical role for the OFC in regulating the ability to adapt behavior when outcome value or identity changes has been largely established, here we present new evidence on the specificity of dependence-induced effects on the computations supporting these processes.

The self-paced nature of our task allowed us to investigate the dynamics of OFC computations made during ongoing decision-making that relies solely on internal representations or retrieval of learned duration contingencies, rather than a reliance on predictive external sensory information. Here, we show with *in vivo* electrophysiology data that the OFC activity was modulated during the lever press itself. OFC activity decreased while mice held down the lever, with OFC activity in air controls resembling a U-shaped pattern during longer presses (Fig. 4). The continuous nature of holding down the lever revealed an activity pattern of an initial decrease in activity relative to baseline, that subsequently increases before the release of the lever press and before when outcomes are expected. The increase in activity before lever press release may correspond to a greater confidence in outcome delivery, something previously shown for OFC activity in cued tasks (Kepecs et al., 2008; Masset et al., 2020). Another possibility stems from prior data from OFC lesioned patients suggesting a potential role for OFC activity in evaluating the passing of time (Berlin et al., 2004; Berlin and Rolls, 2004). Considering reports of increased OFC activity during actions and its association with compulsive action phenotypes similar to those seen in drug studies (Milad and Rauch, 2012; Pauls et al., 2014; Robbins et al., 2019; Lüscher et al., 2020), another hypothesis could be that we would observe larger increases in OFC activity at some point during the duration of lever presses and that CIE may enhance this. Overall, prior alcohol exposure had very minor effects on duration-related activity patterns, although the increase before lever press release was absent in CIE mice. Thus, future experiments aimed at investigating the above hypotheses are warranted.

The dichotomy of CIE effects on the different behavioral components of our task suggests a combination of OFC circuitry changes that could manifest in a variety of ways. In the future, it will be of interest to examine how CIE perturbs OFC function and output that relies on information received by interconnected structures. For example, chronic alcohol exposure and withdrawal may be perturbing the excitability and transmission of local OFC circuitry via cell-type-specific changes (Badanich et al., 2013; McGuier et al., 2015; Nimitvilai et al., 2017; Renteria et al., 2018), such that the integration of incoming information from other associative regions necessary to guide decision-making is disrupted. Additional difficulties in parsing the effects of alcohol dependence on decision-making processes arise from a fundamental lack of structural and functional input-output mapping of the highly complex neural circuits that support decision-making. While in general the areas projecting to OFC have been identified, the relative proportions of inputs across brain regions, as well as the connectivity, strength, and pattern of inputs onto excitatory and inhibitory OFC populations is unknown in naive circumstances, much less following alcohol dependence. It may be that alcohol dependence results in a redistribution of inputs across OFC excitatory and inhibitory populations and/or alters input transmission onto OFC circuits, thereby altering their ability to contribute to decision-making. We should note that it is not clear whether effects observed on neural activity in the present study differ depending on sex. Because of difficulties in female mice maintaining and carrying electrodes and associated head-caps, we were not powered in our *in vivo* recording experiments to examine whether there were any sex differences in our neural data that could differentially mediate contingency and expected outcome value control (Barker et al., 2010).

As with all brain areas, the capacity to contribute to decision-making computations is going to depend on the afferent inputs as well as local capabilities. Alcohol dependence is likely to affect both across the brain. Here, we identified some of the complexity in how OFC’s contributions to decision-making computations are altered following alcohol exposure. These findings will hopefully shed light on the behavioral and OFC-based perturbations previously reported and provide insight into the therapeutic treatment of alcohol dependence.

## Synthesis

Reviewing Editor: Laura Bradfield, University of Technology Sydney Decisions are customarily a result of the Reviewing Editor and the peer reviewers coming together and discussing their recommendations until a consensus is reached. When revisions are invited, a fact-based synthesis statement explaining their decision and outlining what is needed to prepare a revision will be listed below. The following reviewer(s) agreed to reveal their identity: Jacqueline Barker, Christopher Lapish.

The reviewers and myself both feel very positively about this mansucript. There are a few minor comments from each reviewer to be addressed. Please pay particular attention to Figure 2E which both reviewers found confusing, and please include more information about sample size numbers, how many of the cohorts are female etc. in accordance with both reviewers’ comments.

Specific reviews are attached:

Reviewer #1

This very well-written manuscript describes in vivo recording data from a mouse model of chronic intermittent ethanol exposure. The authors observe that, consistent with similar tasks reported in the literature, ethanol dependence via CIE disrupts goal-directed actions. They observe that this is accompanied by increased OFC activity surrounding the action, but reduced OFC activity at outcome information. I want to emphasize that their well-annotated code, highly replicable methodology accompanying a set of interesting data was a joy to read. My comments can all be addressed with textual/graphical edits.

1. While it would be of value to observe the impact of modulated OFC activity during these task epochs on associated behaviors, I do not think that these experiments are necessary for publication of the manuscript. That said, some consideration of the other potential contributions of OFC activity in this task within the discussion would be helpful to aid the reader in identifying potential alternative interpretation and in disentangling these considerations in the absence of additional behavioral/mechanistic experiments. In particular, given that this task requires both inhibitory control and interval timing in the sustained pressing, some discussion on the role of OFC/ impact chronic ethanol on these aspects would be of value.

2. Figure 2E is a bit of a challenge to interpret - the presentation of all possible permutations with colors that are not paired in a meaningful way (i.e., do not translate from the Air to CIE groups) made it very cumbersome to interpret. I’m not sure of the best way to present this information - perhaps breaking into multiple panels to show, e.g., all onset neurons and % of onset neurons that were modulated in additional task components.

3. While I appreciate the transparency surrounding the lack of data in female mice, it would be helpful to appreciate whether, at least numerically if not statistically, differences in behavior (where a greater number of females are included) or neurophysiology were observed, which group the female mouse was in, what % of units were derived from the female mouse, etc.

Reviewer #2

The manuscript is extremely well written and clear (except for a couple of points). I have a couple of questions though:

Why are the rewarded lever presses so low (∼25%). This raises concerns that the animals haven’t mastered the task. How might this influence your interpretation of the data?

Not clear how the normalized response rate was calculated. Please explain more clearly.

Figure 2E is extremely difficult to parse, but I guess this might be b/c there are no differences. I spent a lot of time looking at it. Perhaps this can be reworked in some way?

There is a relatively low number of units in the study leaving open the possibility that one or a few animals are the major contributor. Can you describe the number of units/animal in your data set?

What is the dashed line in the Figure 4 supplement? Is this the criteria? Should this be on the other axis?

No alcohol drinking data? Was the phenotype validated? CIE is established but it’s also important to contextualize this data set.

## Author Response

Reviewer #1

This very well-written manuscript describes in vivo recording data from a mouse model

of chronic intermittent ethanol exposure. The authors observe that, consistent with

similar tasks reported in the literature, ethanol dependence via CIE disrupts goal directed actions. They observe that this is accompanied by increased OFC activity

surrounding the action, but reduced OFC activity at outcome information. I want to

emphasize that their well-annotated code, highly replicable methodology accompanying

a set of interesting data was a joy to read. My comments can all be addressed with

textual/graphical edits.

1. While it would be of value to observe the impact of modulated OFC activity during

these task epochs on associated behaviors, I do not think that these experiments are

necessary for publication of the manuscript. That said, some consideration of the other

potential contributions of OFC activity in this task within the discussion would be helpful

to aid the reader in identifying potential alternative interpretation and in disentangling

these considerations in the absence of additional behavioral/mechanistic experiments.

In particular, given that this task requires both inhibitory control and interval timing in the

sustained pressing, some discussion on the role of OFC/ impact chronic ethanol on

these aspects would be of value.

We thank the reviewer for their positive assessment of our paper overall, and for this

suggestion. We have adjusted the discussion section accordingly.

2. Figure 2E is a bit of a challenge to interpret - the presentation of all possible

permutations with colors that are not paired in a meaningful way (i.e., do not translate

from the Air to CIE groups) made it very cumbersome to interpret. I’m not sure of the

best way to present this information - perhaps breaking into multiple panels to show,

e.g., all onset neurons and % of onset neurons that were modulated in additional task

components.

We thank the reviewer for this insight and have adjusted Figure 2E to include clarifying

labeling and the corresponding percentages from the figure caption in the legend.

3. While I appreciate the transparency surrounding the lack of data in female mice, it

would be helpful to appreciate whether, at least numerically if not statistically,

differences in behavior (where a greater number of females are included) or

neurophysiology were observed, which group the female mouse was in, what % of units

were derived from the female mouse, etc.

We include number of females included in each experiment in the Methods section

(Mice subsection). In the behavioral sub-cohort, we report no experiment in which a

much greater number of females were included in comparison to males (n = 15, 9

males, 6 females for non-recording experiments). In the recording sub-cohort, we

mention that only one female mouse was included due to headcap implant

complications, and as such cannot report a numerical difference from males given this 2

low sample size. In terms of # of significantly modulated units derived from the single

female mouse in the CIE group, we found an average of 8.5 for lever press onset, 4.5

for lever press offset, and 5 for reinforcement delivery, all within the means and

standard deviation for each event type within the CIE group.

Reviewer #2

The manuscript is extremely well written and clear (except for a couple of points). I have

a couple of questions though:

Why are the rewarded lever presses so low (∼25%). This raises concerns that the

animals haven’t mastered the task. How might this influence your interpretation of the

data?

This task requires animals to learn to generate a lever press that meets an arbitrary

criterion. Hence an animal has to rely on an internal program to perform the appropriate

action in an appropriate range. As mice are not automatons, and we specifically did not

want a task which they can easily master and execute behavior more akin to model free

or relying more on habit strategies. Instead, we wanted to bias engagement of more

evaluative cognitive processes, as demonstrated by outcome devaluation testing. We

do present clear evidence of learning. We did see the % success increase (main effect

of session), total number of lever presses made decrease across session (main effect of

session), as well as shifts in duration distributions across criteria as the duration

increased. This task-difficulty allowed us to investigate the difference between OFC

representations when outcomes occurred versus when they were close to succeeding.

While we did not do extensive behavioral exploration in our current manuscript (we have

another large body in preparation of behavioral and modeling data that clearly show

contributions of learning, history, etc., but not pertinent to the effects of alcohol

dependence on OFC activity), we do present learning-induced shifts in duration

distributions. With the implementation of contingencies, mice shift their lever press

duration peaks and distributions depending on the required duration (e.g., Figure 1

figure supplement A and B). That is, mice would not randomly guess to hold down the

lever for 1.6 secs, but instead have learned a distribution through which they sample. To

note, this is a slightly different task than that used by Yin. Unlike their work, we do NOT

have the lever retracting after a successful press, as it provides a cue for reward.

Further, performance (success) is very similar between our groups but are relying of

differing strategies though.

Not clear how the normalized response rate was calculated. Please explain more

clearly.3

Our normalization was test response rate/average response rate from the last two days

of acquisition. We have adjusted the manuscript to include the following in the

Devaluation Testing subsection under Methods.

Response rate comparisons between Valued and Devaluated days were made by

normalizing each mouse’s test day response rate to the average response rate of their

corresponding last two days of 1600-ms duration criterion sessions using the following

formula:

Figure 2E is extremely difficult to parse, but I guess this might be b/c there are no

differences. I spent a lot of time looking at it. Perhaps this can be reworked in some

way?

We thank the reviewer for this insight and have adjusted Figure 2E to include clarifying

labeling and the corresponding percentages from the figure caption in the legend.

There is a relatively low number of units in the study leaving open the possibility that

one or a few animals are the major contributor. Can you describe the number of

units/animal in your data set?

We now add a report of the average # and SEM of significantly modulated units per

animal per event type Given the multifaceted function of OFC populations, we did not

expect the majority of captured units to adjust their firing relative to event type epochs.

Our data is in line with others for the percentage of units showing significant behavior related modulation. Further, between our groups, we have very similar numbers of

significantly modulated units for all events. For lever press onset (Air = 7.833 {plus minus} 1.862,

CIE = 7.278 {plus minus} 1.834), for lever press offset (Air = 8.056 {plus minus} 1.591, CIE = 7.111 {plus minus} 1.699),

for reinforcement delivery (Air = 6.111 {plus minus} 2.274, CIE = 4.611 {plus minus} 1.330). We found no

evidence of animal outliers using the ROUT method in GraphPad Prism.

What is the dashed line in the Figure 4 supplement? Is this the criteria? Should this be

on the other axis?

Yes, we have adjusted Figure 4B to include labeling for the criteria and have added it to

the y-axis as well.

No alcohol drinking data? Was the phenotype validated? CIE is established but it’s also

important to contextualize this data set.4

We do offer a phenotype validation of CIE in our current manuscript. Prior CIE exposure

has been shown to disrupt goal-directed behavior (Lopez & Becker, 2014; Renteria et

al., 2018; 2020). Further, the CIE procedure has been sufficiently validated at a model

of dependence by numerous studies outside of and within our own lab. Further

validation is outside the scope of the current manuscripts focus on decision-making.

Importantly, introducing another independent behavior would have interfered with the

limited dependence-effect time window in which we wanted to train animals for our task

(21 days after last CIE exposure, as reported by previous studies), thus precluding our

ability to ascertain changes in OFC activity during decision-making following CIE

procedures.
